# The Burden of Thin Melanomas in Tuscany, Italy, 1985–2017: Age- and Sex-Specific Temporal Trends in Incidence and Mortality

**DOI:** 10.3390/cancers16030536

**Published:** 2024-01-26

**Authors:** Gianfranco Manneschi, Adele Caldarella, Saverio Caini, Saverio Checchi, Teresa Intrieri, Alessandra Chiarugi, Paolo Nardini, Giovanna Masala

**Affiliations:** 1 Clinical Epidemiology Unit, Institute for Cancer Research, Prevention and Clinical Network (ISPRO), 50139 Florence, Italy; g.manneschi@ispro.toscana.it (G.M.); a.caldarella@ispro.toscana.it (A.C.); t.intrieri@ispro.toscana.it (T.I.); g.masala@ispro.toscana.it (G.M.); 2 Cancer Risk Factors and Lifestyle Epidemiology Unit, Institute for Cancer Research, Prevention and Clinical Network (ISPRO), 50139 Florence, Italy; 3 Postgraduate School in Hygiene and Preventive Medicine, University of Florence, 50144 Florence, Italy; saverio.checchi@unifi.it; 4 Screening and Secondary Prevention Unit, Institute for Cancer Research, Prevention and Oncological Network (ISPRO), 50139 Florence, Italy; a.chiarugi@ispro.toscana.it (A.C.); p.nardini@ispro.toscana.it (P.N.)

**Keywords:** cutaneous melanoma, Breslow thickness, incidence, mortality, trend, Tuscany, Italy

## Abstract

**Simple Summary:**

We aimed to study whether the burden of disease (incidence and mortality) of thin melanomas (i.e., melanomas thinner than 1 mm at diagnosis) increased in recent decades in Italy as observed in other world countries (e.g., Australia, the USA, and Scandinavian countries). We used data from a population-based cancer registry in Tuscany (Central Italy) and found that, while the incidence of thin melanomas has indeed been increasing steadily in recent years (much more than thick melanomas), the contribution of thin melanomas to mortality has not changed substantially. Moreover, we detected differences between sexes and across age groups in the way the epidemiology of melanoma, and especially thin melanomas, has changed in recent years. These findings likely reflect parallel changes in exposure to UV radiation (the most important environmental risk factor for melanoma) and possibly early diagnosis practices, and they represent a valuable piece of information for informing effective prevention interventions.

**Abstract:**

A steady increase in the incidence and mortality burden correlated to thin melanomas (≤1 mm) has been reported in recent years in some international studies, but there is currently a paucity of data from the Mediterranean area. We aimed to describe the epidemiological characteristics of thin melanoma in Tuscany, Central Italy. A total of 6002 first cutaneous invasive melanomas occurring from 1985 to 2017 were selected for analysis; data were retrieved from the local population-based cancer registry. The standardized incidence rate was 15.0 per 100,000 in the population, higher among men than women (16.5 vs. 14.1). Incidence rates tended to increase over time across all age group-specific population strata, with annual percent changes moderately higher among men (+8.0%) than women (+6.9%), especially among the elderly. Among both sexes and in each age group, the trend toward increasing incidence rates was particularly strong for thin melanomas. Survival was better among women than men across all categories of thickness. Approximately 15% of deaths occurred among patients with thin lesions, with no major temporal changes in recent years. This study contributes to an improved understanding of melanoma epidemiology in Tuscany and underscores the need for primary prevention strategies tackling the growing burden of thin melanomas.

## 1. Introduction

Cutaneous melanoma, a skin cancer caused by a malignancy of melanocytes, is a significant public health concern worldwide, as its incidence has been steadily increasing over the past few decades. In a recent assessment based on the GLOBOCAN 2020 database, the number of new melanoma cases in 2020 was estimated at 325,000, with 57,000 associated deaths. Moreover, the global burden from melanoma is expected to increase in the next decades, with projections reaching up to 510,000 new cases and 96,000 deaths by 2040 [1] Individuals with fair skin types, particularly those of European descent, have a greater susceptibility to melanomas. The countries with the highest burden of melanoma are Australia and New Zealand, followed by Western Europe, Northern America, and Northern Europe [2].

Confirmed prognostic factors for melanoma include the Breslow depth, Clark level, tumor ulceration, tumor location, vascular invasion, and patient gender. The single most important prognostic factor for melanoma is the Breslow depth of the primary tumor [3,4]. Melanoma survival is strongly inversely associated with lesion thickness; thus, thin lesions have a better prognosis than thicker ones. Thick melanomas (>4 mm) have a 20-year survival rate of approximately 50% [5]. Ulceration is a relevant factor as well, since studies consistently report that melanomas with ulceration present a significantly lower survival rate compared to non-ulcerated ones [6]. Characteristics associated with prognosis also include genetic susceptibility, number of mitoses, histopathologic subtype, somatic mutation status, tumor mutational burden, and others [6]. “Thin melanomas” are tumors less than 1 mm thick, according to the Breslow classification, and they usually have a favorable prognosis, presenting a 20-year survival rate of 96% [7]. Thin melanomas comprise the vast majority of all diagnosed melanomas, and they are responsible for the global increasing incidence trend [8,9,10]. Despite their low lethality, the high and still growing incidence rates mean that thin melanomas are generally associated with a very high burden of disease.

Previous publications have shown that approximately 70% of the new cases of malignant melanomas are thin [8] and that a substantial proportion of melanoma deaths may be associated with thin lesions [11,12]. A 2015 Australian study conducted in Queensland showed that a higher number of people die from thin lesions than from thick melanomas (>4 mm) [8]. The increase in melanoma death rates concerns, in particular, the male population and those aged 65 years or older [6,11,12]. This poses both public health questions (e.g., relative to primary and secondary prevention) and clinical dilemmas, given the difficulty of distinguishing thin melanomas that will evolve towards more serious forms from those that will not progress further. Understanding the clinical and pathological features associated with progressive thin melanoma is seen as crucial for accurate risk stratification and optimal patient management [13].

Knowing that the epidemiology of melanoma depends on factors that vary geographically as well as over time in a given setting (e.g., demographic structure of the resident population, solar irradiance and UV index, age- and sex-specific patterns of exposure to UV radiation, and the implementation of prevention campaigns), it is critically important to analyze local data to gain an in-depth understanding of the epidemiological landscape. The studies existing to date that specifically focus on thin melanomas (in terms of incidence, time trends, and mortality burden) were mostly performed in Australia, the USA, and Scandinavian countries, while much less is known on this subject for Mediterranean countries. In this work, we studied the epidemiology of melanoma, focusing specifically on thin melanoma in Tuscany, Central Italy, using data from a population cancer registry for the period 1985–2017.

## 2. Materials and Methods

### 2.1. The Tuscany Cancer Registry

The data used in this article were retrieved from the Tuscany Cancer Registry (RTT; https://www.ispro.toscana.it/rtt), a population-based cancer registry active in Central Italy since 1985. At its foundation, the RTT covered only two provinces in Tuscany (Florence and Prato, population ≈ 1.2 million); in 2013, it was extended to the whole region (≈3.5 million inhabitants). The RTT draws upon multiple sources of information to collate information on all incident cancer cases in the population residing in the area; these include hospital discharge records, death certificates, pathology reports, and other sources. The RTT has been part of the Italian Network of Cancer Registries (AIRTUM) since the latter was established in 1997 and has participated in several collaborative studies involving other cancer registries in Italy.

We selected for inclusion in this study all first primary cutaneous melanoma cases that were registered between 1985 and 2017 (with the exception of the year 2012, when the activities of the register were temporarily discontinued). For consistency, the analysis was limited to the area of Florence and Prato, where the register has been active since 1985 (for conciseness, however, we will use the term Tuscany in what follows). Melanoma cases with missing information on Breslow thickness at diagnosis were excluded from the analysis.

### 2.2. Statistical Methods

Incidence rates per 100,000 of the population were standardized by age via the direct method using the European Standard Population 2013. Trends in standardized incidence rates were investigated by joinpoint regression analyses and described by using annual percent changes (APCs). The joinpoint model involves fitting a series of straight lines on a log scale to the trends in the annual age-standardized rates, as described in detail elsewhere [14]. APC corresponds to the slope of the line segment; APC values significantly different from zero are interpreted as evidence that the trend in rates changed over time. Melanomas were categorized into the three following subgroups according to Breslow thickness at diagnosis: ≤1 (thin), >1–4, and >4 mm. The results were presented (overall and by Breslow thickness category) for the whole population and separately defined within strata by sex and age group (<40, 40–64, and ≥65 years). We calculated and plotted the observed overall survival of melanoma patients according to sex and Breslow thickness category. Concerning mortality data, the study time frame was divided into four periods: 1985–1990, 1991–2000, 2001–2010, and 2011–2017 (i.e., each starting or ending, depending on data availability and the first or last year of a decade). We calculated the total number of deaths among melanoma patients that occurred in each period as well as their median number per year (the latter was intended to ease comparison across periods, as the number of years included in each period differed due to data availability). We then calculated the proportion of deaths that occurred among patients whose melanoma was ≤1, >1–4, and >4 mm at diagnosis, overall and by sex and age group (given the low numbers of deaths in the younger age group, the <40 and 40–64 age groups were merged, and the resulting category was compared to melanoma patients aged ≥65 years). Statistical analyses were performed using the SEER*Stat VERSION 8.3.5 software. All statistical tests were two-sided, and the *p*-value was considered statistically significant when below 0.05.

## 3. Results

A total of 7062 first primary cutaneous melanomas were diagnosed in Tuscany during 1985–2017 (except 2012, for which no data were available), of which 6002 (85.0%) had information on Breslow thickness at diagnosis and were included in the analyses (the proportion of cutaneous melanomas whose Breslow thickness was unknown did not vary over time; the results were not shown). These were almost equally divided between men (3012, 50.2%) and women (2990, 49.8%) (Table 1). The trunk was the most frequent anatomical location (29.9%), followed by the lower (19.4%) and upper (10.5%) limbs (for 33.1% of cases, this information was not available) (Table S1). In terms of histological subtype, 71.3% of the included cases were superficial spreading melanomas, with nodular melanoma being the second most frequent occurrence (7.5%) (Table S1). The overall standardized incidence rate was 15.0 per 100,000 of the population, somewhat higher among men than women (16.5 vs. 14.1 per 100,000). Men and women differed in terms of age at diagnosis; the proportion of those diagnoses at <40, 40–64, or ≥65 years of age was 12.8%, 46.5%, and 40.7% among men and 20.3%, 46.3%, and 33.4% among women. Consistently, the standardized incidence rate was lower among males than females in the youngest age group (<40 years: 6.6 vs. 4.2 per 100,000 of the population), similar between sexes in the 40–64 years-old group (22.1 vs. 20.4 per 100,000 men and women, respectively), and much larger among men than women among older individuals (36.4 vs. 21.2 per 100,000) (Table 1).

Trends in incidence rates also differed substantially between genders and across age groups. Overall, there was a trend toward increasing incidence rates over time across all sex- and age group-specific population strata during the study period. However, APCs were always greater in males than females, with a narrow gap in the younger age group (+5.8% vs. 5.4%), which then widened in the 40–64 age group (+6.0% vs. +4.8%) and reached the maximum difference among older individuals (+7.4% vs. +4.9%) (Table 1).

The results of the analyses stratified by Breslow thickness are reported in Table 2 for men and Table 3 for women (and in Table S2 for the whole study population). The percentage of cutaneous melanomas that were ≤1, >1–4, and >4 mm thick at diagnosis was 62.1%, 27.9%, and 10.0% among men and 65.1%, 26.8%, and 7.3% among women. Sex- and melanoma thickness-specific temporal trends in melanoma incidence rates are reported in Figures S1 and S2 (for men and women, respectively). As more thoroughly described in a previous article [15], the median Breslow thickness decreased significantly over time among both sexes for melanomas comprised within the ≤1 and >1–4 mm thickness categories, while there was no significant time trend in the median thickness of melanomas >4 mm at diagnosis.

Among both sexes and in each age group, the trend toward increasing incidence rates was particularly strong for thin melanomas (≤1 mm). In particular, the APC for thin melanomas was +8.3%, +7.8%, and +10.3% (for those aged <40, 40–64, and ≥65 years at diagnosis) among men, constantly above the corresponding values calculated among women (+6.0%, +6.6%, and +8.0%, respectively) and with no evidence that the upward trend had slowed, stopped, or reversed during the observation period (Table 2 and Table 3).

The picture was comparatively less uniform for thicker melanomas. The standardized incidence rates of melanomas >1–4 mm thick moderately increased over time (APC within 5%, for men aged 40–64 or ≥65 years, and for women <40 or ≥65 years old) or remained flat (men in the youngest age group and women aged 40–64 years) during the study period (Table 2 and Table 3). Concerning melanomas >4 mm thick, in individuals of both sexes younger than 40 years and among women aged 65 years or older, an initial period of growing incidence rates was followed by a reversal of the trend, with a slope in incidence rates that was generally steeper although shorter in duration (the change points occurred between 2004 and 2009). In the remaining demographic segments, the trend in incidence rates over time was either inexistent (people of both sexes aged 40–64 years) or moderately increasing (APC +3.8% among men aged ≥65 years) (Table 2 and Table 3).

**Figure 1:**
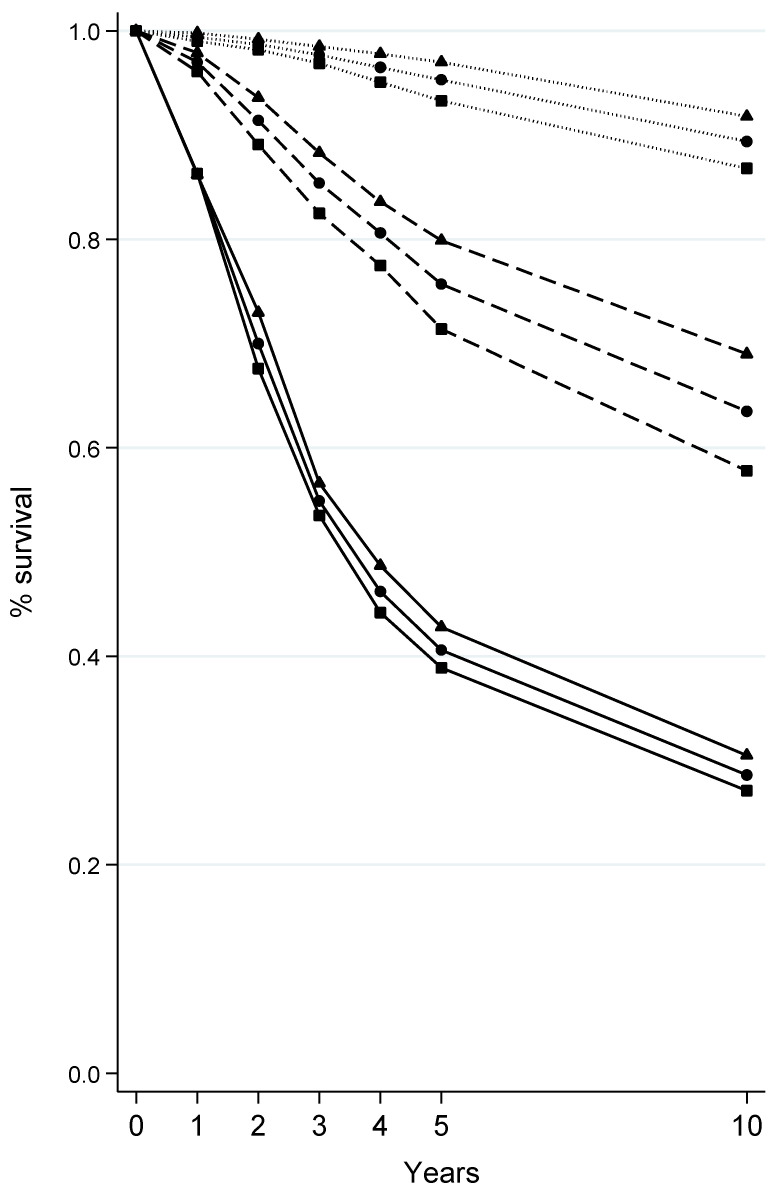
Survival at 1, 2, 3, 4, 5, and 10 years from the diagnosis of cutaneous melanoma according to sex and Breslow thickness at diagnosis. Tuscany, Central Italy, 1985–2017. Only cutaneous melanomas with known Breslow thickness at diagnosis were included. Sex: ■ males; ▲ females; ● both sexes combined. Breslow thickness at diagnosis: ∙∙∙∙ <1 mm; --- 1–4 mm; ― >4 mm.

Survival rates of melanoma at 1, 2, 3, 4, 5, and 10 years after diagnosis according to sex and Breslow thickness at diagnosis are depicted in Figure 1. By and large, survival rates fell steeply with increasing Breslow thickness; for instance, the proportion of melanoma patients who were still alive 5 years after diagnosis was 95.3%, 75.7%, and 40.6% for lesions that were ≤1, >1–4, and >4 mm in thickness (Table S3). Moreover, survival was consistently better among women than men, with the gap between sexes being larger for melanomas >1–4 mm (e.g., at 5 years upon diagnosis: 71.4% vs. 79.9%) than for thinner (≤1 mm: 93.3% vs. 97.0%) and thicker (>4 mm: 38.9% vs. 42.8%) melanomas (Figure 1 and Table S3).

Table 4 reports the number of melanoma patients who died in each of the four decades encompassed (fully or partially) by our observation period, both overall and stratified by sex, age group (< vs. ≥65 years), and Breslow thickness categories. The median number of deaths (per year) among <65-year-old melanoma patients was rather stable during 1985–2010 and decreased thereafter, while in the older population (≥65 years), the median number of deaths per year rose over time to peak in the first decade of the 21st century, with a reversal of the trend in more recent years (from 2011 onwards). As detailed above, our interest was in quantifying the proportion of all deaths among melanoma patients that occurred among those with lesions ≤1, >1–4, and >4 mm thick and in monitoring how these proportions evolved over time during the study period.

When ignoring the breakdown by age, a clear pattern emerged, evident among both sexes, according to which the contribution of lesions >1–4 mm thick to the total number of deaths among melanoma patients decreased over time (from 59.7% and 59.6% in 1985–1990 to 40.8% and 44.7% in 2011–2017 among males and females, respectively), while an opposite trend was observed for melanomas thicker than 4 mm (from 28.4% and 21.3% in 1985–1990 to 44.7% and 42.1% in 2011–2017 among males and females, respectively) (Table 4). Melanomas ≤1 mm thick made up a minority share of deaths among melanoma patients, with their proportion moderately on the rise among men (from 11.9% to 14.5%), while decreasing by nearly one third over the study period among women (from 19.1% to 13.2%). In the age-stratified analysis, the pattern described above applied to deaths in melanoma patients younger than 65, while among older subjects (≥65 years), the share of deaths among patients with lesions >4 mm remained stable among men and increased moderately among women (from 36.8% to 43.5%), and the contribution of patients with lesions ≤1 mm to the total number of deaths tended to increase over time among men and among women as well (although not linearly) (Table 4).

In the last interval of the observation period (2011–2017), individuals with melanomas whose thickness was >1–4 or >4 mm both comprised 40% to 50% of the number of total deaths that occurred among melanoma patients, irrespective of sex and age group, while the contribution of thin melanomas (≤1 mm) ranged between 8.7% and 20.0%, depending on patients’ demographics.

## 4. Discussion

We studied the epidemiology of cutaneous melanomas in Tuscany, Central Italy, during 1985–2017, with a particular focus on Breslow thickness and with the ultimate goal of gaining a better understanding of the disease burden of thin melanomas (in terms of incidence, survival, morality, and respective temporal trends). Thin melanomas (≤1 mm) had the highest incidence rates and the steepest and most consistent growth trend over the study period. There were noteworthy sex-based differences in melanoma outcomes, with consistently better survival rates among women regardless of thickness category, which warrants further investigation into potential sex-specific factors influencing melanoma prognosis. Finally, the number of melanoma patients that passed away per year showed an age group-specific time trend, yet the proportion of total deaths among melanoma patients that occurred among those with thin melanomas did not exhibit any substantial change over the study period, unlike that of thick melanomas (>4 mm), whose relative weight increased over time.

Our findings corroborate previous research indicating a notable increase in the incidence rates of thin melanoma over recent decades. Similar trends have been observed in many countries around the world, and they are common, for example, to most European countries [16]. Of note, studies conducted in Australia [12], Sweden [6], Denmark [17], and the USA [11] have reported largely similar results, despite the fact that the most important characteristics affecting melanoma epidemiology, namely phototype and habits of exposure to sunlight and artificial UV radiation, are likely to be distributed differently than they are in Italy. The observed upward trend is most likely multifactorial, influenced by complex changes over time in terms of patterns of exposure to UV radiation, heightened public awareness, advances in diagnostic technologies, and skin cancer screening practices. In particular, data on temporal trends in the prevalence of exposure to UV radiation (the main environmental risk factors for melanoma) show considerable geographic variability; while sun protection behaviors seem to have gradually become more widespread over time in Australia and the USA [18,19], the picture is far less optimistic in Europe (including Italy), where the proportion of melanoma cases potentially preventable through primary prevention remains very high [20,21,22,23]. Data on the prevalence and temporal trends of self-skin examination are even more sparse, and the impact of these preventive behaviors on the rising melanoma trends remains unclear. By and large, the observed trends necessitate ongoing surveillance and research to identify the underlying behavioral determinants, and, in particular, the higher incidence rates among men in the older age group warrant focused attention, as well as the much higher incidence of thin melanomas among young (<40 years) women compared to men of the same age.

The evidence regarding sex-based differences in the survival of melanoma patients is established and robust. Women have a survival advantage over men when diagnosed with melanoma, regardless of known prognostic factors like tumor stage, age at diagnosis, and others [24,25]. In particular, women with localized melanomas (which make the majority of cases at diagnosis) have a reduced risk of progression as well as lymph node and visceral metastasis [26]. Interestingly, sex differences in melanoma extend to the response to most recently available systemic treatments, namely immune therapy (for which genetic bases are starting to be unveiled) and targeted therapy [27,28,29]. Sex might therefore be a factor to be considered (along with tumor thickness and stage and other prognostic factors) in the management of melanoma patients.

The temporal trend in the number of deaths among melanoma patients differed somewhat between age groups; of particular interest was the analysis of trends in mortality by category of Breslow thickness. In particular, the stability in the proportion of total deaths associated with thin melanomas (despite the steep rise in incidence rates) is an intriguing finding and runs counter to the expectations derived from previous publications [11,12]. Thin melanoma inherently requires a longer timeframe to exert an influence on mortality rates, owing to its superior prognostic characteristics and extended progression period relative to thicker melanomas. Thus, the anticipated impact of increased incidence on mortality might still have to manifest, suggesting that it is too premature to discern such effects. This hypothesized time lag might be further accentuated by the trend towards a decrease in the thickness of thin melanomas (≤1 mm) that occurred during the observation period, and the parallel finding for melanomas >1–4 mm thick may help explain the progressive reduction in the share of total deaths caused by melanomas in that thickness category (which may also partly follow improvements in the management or early detection of moderately thick melanomas). The increasing trend in deaths attributed to melanomas exceeding 4 mm at diagnosis (especially among younger age groups) underscores the challenges associated with late-stage presentations and signals a worrying unmet health need in the population of Tuscany. Finally, thickness-specific incidence trends of cutaneous melanoma in Tuscany mirror those reported in other world areas with predominantly fair-skinned populations, and it is therefore to be expected that the mortality burden of thin melanomas will also increase in the coming decades, as already extensively documented in other countries [30].

The findings of this study contribute valuable insights into the incidence and mortality patterns of melanoma in the Tuscany region, shedding light on crucial aspects that can inform both public health strategies and clinical practice. From a public health perspective, understanding the variations in incidence and the survival impact of early-stage melanoma is important to optimize prevention and ensure early diagnosis along with appropriate treatment and follow-up. Heightened awareness surrounding melanoma, especially thin lesions, coupled with education on sun-safe practices and early detection, may lead to substantial reduction in the burden of melanoma. Additionally, our study suggests the importance of culturally sensitive programs aimed at populations at increased melanoma risk. In this regard, men aged ≥65 years were found to have higher incidence rates than women in the same age group, while the opposite was true among <40-year-old individuals. Moreover, male melanoma patients have substantially worse survival than females across Breslow thickness categories. Sex-specific habits of exposure to UV radiation and response to specific treatments, as well as hormonal factors and genetic predispositions, may all contribute to discernible patterns of melanoma among men and women. Recognizing and addressing sex differences in melanoma epidemiology is therefore paramount for developing comprehensive and more effective approaches to prevention, diagnosis, and treatment and tackle disparities in incidence and mortality. Moreover, with reference to thin melanomas, it is critically important to improve our ability to discriminate between the few that have potential for progression and many that do not (and may actually be due to overdiagnosis [31]), as this would ensure that subjects with potentially thin melanomas could be managed appropriately in terms of therapy and subsequent follow-up.

Our study has several strengths. The Tuscan Cancer Registry, operational since 1985 and integrated into the national network, upholds a well-established methodology and has served as the foundation for numerous prior publications, including concerning melanomas [15,32,33]. Notably, the population of the Tuscany region and its age distribution varied only marginally over the past 20 years, easing the interpretation of temporal trends of incidence and mortality data. It is also essential to acknowledge the limitations of our study. The proportion of cutaneous melanomas with unknown thickness was neither major nor negligible (15%), curbing the strength and robustness of our results. While there is no a priori reason to suspect that the absence of data on thickness is informative (i.e., not at random), this cannot be formally assessed. A larger study size would have made it possible to further split the 1–4 mm category into 1–2 mm and 2–4 mm subgroups, thus facilitating the identification of nuanced trends in incidence and mortality within these subgroups. Finally, the completeness of information was limited with regard to other variables with clinical relevance, which, therefore, could not be brought into the analysis.

## 5. Conclusions

In conclusion, we found that thin cutaneous melanomas grew in incidence during 1985–2017 in Tuscany more than thick lesions, without this having any evident effect so far in terms of mortality. Moreover, the epidemiology of melanoma, including that of thin lesions, presented important specificities with regard to sex and age groups. By and large, the observed patterns have important public health implications, as they underscore the imperative for prevention strategies aimed at containing the growing burden of this malignancy. In particular, comprehensive and targeted intervention programs emphasizing a community awareness of melanoma risk factors and education on sun-safe behaviors are of paramount importance in the attempt to tackle and possibly reverse the growing incidence trends of this malignancy in Tuscany.

## Figures and Tables

**Table 1:** The number of incident cutaneous melanoma cases, standardized incidence rate (with 95% confidence intervals, CI), and annual percent changes (APC) in different periods, stratified by sex and age group. Tuscany, Central Italy, 1985–2017. Only cutaneous melanomas with known Breslow thickness at diagnosis were included.

Age Group	Melanoma Cases (N)	Standardized Incidence Rate	95% CI	Trend 1		Trend 2 ^(a)^	
				Years	APC	Years	APC ^(a)^
Males							
<40 years	387	4.2	(3.8–4.6)	1985–2017	+5.8%		
40–64 years	1400	22.1	(20.9–23.2)	1985–2017	+6.0%		
≥65 years	1225	36.4	(34.4–38.6)	1985–2017	+7.4%		
All ages	3012	16.5	(15.9–17.1)	1985–2009	+8.0%	2009–2017	+3.0%
Females							
<40 years	607	6.6	(6.0–7.1)	1985–2017	+5.4%		
40–64 years	1383	20.4	(19.3–21.5)	1985–2017	+4.8%		
≥65 years	1000	21.2	(19.9–22.6)	1985–2017	+4.9%		
All ages	2990	14.1	(13.6–14.6)	1985–2007	+6.9%	2007–2017	ns
Both sexes							
<40 years	994	5.4	(5.0–5.7)	1985–2017	+5.7%		
40–64 years	2783	21.2	(20.4–22.0)	1985–2017	+5.4%		
≥65 years	2225	27.5	(26.4–28.7)	1985–2017	+6.3%		
All ages	6002	15.0	(14.6–15.4)	1985–2009	+7.2%	2009–2017	ns

ns: not significant, ^(a)^ only shown when statistically significant.

**Table 2:** The number of incident cutaneous melanoma cases among males, standardized incidence rate (with 95% confidence intervals, CI), and annual percent changes (APC) in different periods, stratified by age group. Tuscany, Central Italy, 1985–2017. Only cutaneous melanomas with known Breslow thickness at diagnosis were included.

Age Group	Melanoma Cases (N)	Standardized Incidence Rate	95% CI	Trend 1		Trend 2 ^(a)^	
				Years	APC	Years	APC
<40 years							
≤1 mm	267	2.9	(2.5–3.2)	1985–2017	+8.3%		
>1–4 mm	100	1.1	(0.9–1.3)	1985–2017	ns		
>4 mm	20	0.2	(0.1–0.3)	1985–2004	+12.3%	2004–2017	−12.4%
40–64 years							
≤1 mm	937	14.7	(13.8–15.7)	1985–2017	+7.8%		
>1–4 mm	377	6.0	(5.4–6.6)	1985–2017	+2.6%		
>4 mm	86	1.4	(1.1–1.7)	1985–2017	ns		
≥65 years							
≤1 mm	668	19.6	(18.1–21.1)	1985–2017	+10.3%		
>1–4 mm	363	10.8	(9.7–12.0)	1985–2017	+4.6%		
>4 mm	194	6.0	(5.2–7.0)	1985–2017	+3.8%		
All ages							
≤1 mm	1872	10.1	(9.7–10.6)	1985–2009	+11.2%	2009–2017	+4.4%
>1–4 mm	840	4.6	(4.3–4.9)	1985–2017	+3.3%		
>4 mm	300	1.7	(1.5–1.9)	1985–2004	+4.5%	2004–2017	ns

ns: not significant, ^(a)^ only shown when statistically significant.

**Table 3:** The number of incident cutaneous melanoma cases among females, standardized incidence rate (with 95% confidence intervals, CI), and annual percent changes (APC) in different periods, stratified by age group. Tuscany, Central Italy, 1985–2017. Only cutaneous melanomas with known Breslow thickness at diagnosis were included.

Age Group	Melanoma Cases (N)	Standardized Incidence Rate	95% CI	Trend 1		Trend 2 ^(a)^	
				Years	APC	Years	APC
<40 years							
≤1 mm	464	5.0	(4.5–5.5)	1985–2017	+6.0%		
>1–4 mm	127	1.4	(1.2–1.7)	1985–2017	+3.0%		
>4 mm	16	0.2	(0.1–0.3)	1985–2009	+6.6%	2009–2017	−19.8%
40–64 years							
≤1 mm	966	14.3	(13.4–15.2)	1985–2017	+6.6%		
>1–4 mm	350	5.2	(4.6–5.7)	1985–2017	ns		
>4 mm	67	1.0	(0.8–1.2)	1985–2017	ns		
≥65 years							
≤1 mm	532	11.6	(10.6–12.6)	1985–2017	+8.0%		
>1–4 mm	331	6.9	(6.2–7.7)	1985–2017	+1.4%		
>4 mm	137	2.7	(2.3–3.2)	1985–2007	+6.4%	2007–2017	−9.5%
All ages							
≤1 mm	1962	9.4	(9.0–9.8)	1985–2007	+9.5%	2007–2017	ns
>1–4 mm	808	3.7	(3.5–4.0)	1985–2017	+1.6%		
>4 mm	220	0.9	(0.8–1.1)	1985–2006	+6.4%	2006–2017	−7.1%

ns: not significant, ^(a)^ only shown when statistically significant.

**Table 4:** The number of deaths by cutaneous melanomas (median and total, rounded to the lower integer) and proportion by Breslow thickness at diagnosis by period, according to sex and age group. Tuscany, Central Italy, 1985–2017.

Group	1985–1990	1991–2000	2001–2010	2011–2017	Group	1985–1990	1991–2000	2001–2010	2011–2017	Group	1985–1990	1991–2000	2001–2010	2011–2017
Men					Women					Both Sexes				
<65 years, median (total)	7 (50)	7 (68)	7 (76)	4 (21)	<65 years, median (total)	6 (28)	5 (53)	5 (54)	3 (15)	<65 years, median (total)	14 (78)	12 (121)	13 (130)	5 (36)
≤1 mm	16.00%	13.20%	15.80%	14.30%	≤1 mm	32.10%	22.60%	16.70%	20.00%	≤1 mm	21.80%	17.40%	16.20%	16.70%
>1–4 mm	62.00%	64.70%	52.60%	42.90%	>1–4 mm	57.10%	54.70%	50.00%	40.00%	>1–4 mm	60.30%	60.30%	51.50%	41.70%
>4 mm	22.00%	22.10%	31.60%	42.90%	>4 mm	10.70%	22.60%	33.30%	40.00%	>4 mm	17.90%	22.30%	32.30%	41.70%
≥65 years, median (total)	3 (17)	5 (53)	9 (86)	8 (55)	≥65 years, median (total)	3 (19)	5 (53)	7 (72)	3 (23)	≥65 years, median (total)	6 (36)	9 (106)	16 (158)	10 (78)
≤1 mm	0.00%	9.40%	11.60%	14.50%	≤1 mm	0.00%	11.30%	15.30%	8.70%	≤1 mm	0.00%	10.40%	13.30%	12.80%
>1–4 mm	52.90%	56.60%	45.30%	40.00%	>1–4 mm	63.20%	56.60%	50.00%	47.80%	>1–4 mm	58.30%	56.60%	47.50%	42.30%
>4 mm	47.10%	34.00%	43.00%	45.50%	>4 mm	36.80%	32.10%	34.70%	43.50%	>4 mm	41.70%	33.00%	39.20%	44.90%
All ages, median (total)	10 (67)	12 (121)	16 (162)	12 (76)	All ages, median (total)	9 (47)	10 (106)	12 (126)	5 (38)	All ages, median (total)	20 (114)	22 (227)	28 (288)	16 (114)
≤1 mm	11.90%	11.60%	13.60%	14.50%	≤1 mm	19.10%	17.00%	15.90%	13.20%	≤1 mm	14.90%	14.10%	14.60%	14.00%
>1–4 mm	59.70%	61.20%	48.80%	40.80%	>1–4 mm	59.60%	55.70%	50.00%	44.70%	>1–4 mm	59.60%	58.60%	49.30%	42.10%
>4 mm	28.40%	27.30%	37.70%	44.70%	>4 mm	21.30%	27.40%	34.10%	42.10%	>4 mm	25.40%	27.30%	36.10%	43.90%

## Data Availability

The data presented in this study are available upon reasonable request from the corresponding author. The data are not publicly available due to privacy limitations.

## Supplementary Materials

The following supporting information can be downloaded at: https://www.mdpi.com/article/10.3390/cancers16030536/s1, Table S1: Distribution of incident cutaneous melanoma included in the analyses in terms of anatomical location and histological subtype. Tuscany, Central Italy, 1985–2017. Only cutaneous melanomas with known Breslow thickness at diagnosis (n = 6002) were included. Table S2: Number of incident cutaneous melanoma cases among both sexes combined, standardized incidence rate (with 95% confidence intervals, CI), and annual percentage changes (APC) in different periods, stratified by age group. Tuscany, Central Italy, 1985–2017. Only cutaneous melanomas with known Breslow thickness at diagnosis were included; Table S3: Survival at 1, 2, 3, 4, 5, and 10 years from diagnosis (with 95% confidence intervals, CI) of cutaneous melanoma according to sex and Breslow thickness at diagnosis. Tuscany, Central Italy, 1985–2017. Only cutaneous melanomas with known Breslow thickness at diagnosis were included. Figure S1: Annual age-standardized melanoma incidence rates among men, overall and according to Breslow thickness at diagnosis (≤1, >1–4, >4 mm) and corresponding temporal trends (calculated via joinpoint analysis—see Section 2.2 for details). Tuscany, Central Italy, 1985–2017. Only cutaneous melanomas with known Breslow thickness at diagnosis were included. Figure S2: Annual age-standardized melanoma incidence rates among women, overall and according to Breslow thickness at diagnosis (≤1, >1–4, >4 mm), and corresponding temporal trends (calculated via joinpoint analysis—see Section 2.2 for details). Tuscany, Central Italy, 1985–2017. Only cutaneous melanomas with known Breslow thickness at diagnosis were included.
