# Supplementary material for: The Burden of Thin Melanomas in Tuscany, Italy, 1985–2017: Age- and Sex-Specific Temporal Trends in Incidence and Mortality
Source: Cancers (Basel). 2024 Jan 26;16(3):536. doi: 10.3390/cancers16030536 (PMC10854552; doi:10.3390/cancers16030536)

Table S1. Distribution of incident cutaneous melanoma included in the analyses in terms of anatomical location and histological subtype. Tuscany, central Italy, 1985-2017. Only cutaneous melanoma with known Breslow thickness at diagnosis (n=6,002) were included.

**Anatomical location**		
Trunk	1,794	29.9%
Head and neck	416	6.9%
Upper limbs	631	10.5%
Lower limbs	1,164	19.4%
Overlapping areas	13	0.2%
Not specified	1,984	33.1%
**Histological subtype**		
Superficial spreading melanoma	4,281	71.3%
Nodular melanoma	453	7.5%
Lentigo maligna	192	3.2%
Acral lentiginous melanoma	185	3.1%
Other subtypes	196	3.3%
Not specified	695	11.6%

Table S2. Number of incident cutaneous melanoma cases among both sexes combined, standardized incidence rate (with 95% confidence intervals, CI), and annual percent changes (APC) in different periods, stratified by age group. Tuscany, central Italy, 1985-2017. Only cutaneous melanoma with known Breslow thickness at diagnosis were included.

Age group	Melanoma cases (N)	Standardized incidence rate	95% CI	Trend 1		Trend 2	
				Years	APC	Years	APC [(a)]
**<40 years**							
≤1 mm	731	3.9	(3.6 - 4.2)	1985-2017	+ 6.7%		
>1-4 mm	227	1.2	(1.1 - 1.4)	1985-2017	+ 2.9%		
>4 mm	36	0.2	(0.1 - 0.3)	1985-2004	+ 11.4%	2004-2017	- 11.7%
**40-64 years**							
≤1 mm	1,903	14.5	(13.8 - 15.1)	1985-2017	+ 7.3%		
>1-4 mm	727	5.5	(5.1 – 6.0)	1985-2017	+ 1.8%		
>4 mm	153	1.2	(1.0 - 1.4)	1985-2017	ns		
**≥65 years**							
≤1 mm	1,200	14.9	(14.1 - 15.8)	1985-2017	+ 9.3%		
>1-4 mm	694	8.5	(7.9 - 9.2)	1985-2017	+ 3.2%		
>4 mm	331	4.0	(3.6 - 4.5)	1985-2005	+ 5.6%	2005-2017	- 7.1%
**All ages**							
≤1 mm	3,834	9.6	(9.3 - 9.9)	1985-2009	+ 9.9%	2009-2017	+ 3.0%
>1-4 mm	1,648	4.1	(3.9 - 4.3)	1985-2017	+ 2.4%		
>4 mm	520	1.3	(1.2 - 1.4)	1985-2007	+ 4.7%	2006-2017	ns

ns: not significant

[(a)] only shown when statistically significant

Table S3. Survival at 1, 2, 3, 4, 5, and 10 years from diagnosis (with 95% confidence intervals, CI), of cutaneous melanoma according to sex and Breslow thickness at diagnosis. Tuscany, central Italy, 1985-2017. Only cutaneous melanoma with known Breslow thickness at diagnosis were included.

Sex	Age group	Breslow thickness	1 year		2 years		3 years		4 years		5 years		10 years	
			survival	95% CI	survival	95% CI	survival	95% CI	survival	95% CI	survival	95% CI	survival	95% CI
any	any	≤1 mm	0.994	(0.991; 0.996)	0.987	(0.983; 0.990)	0.977	(0.972; 0.982)	0.965	(0.958; 0.971)	0.953	(0.945; 0.960)	0.894	(0.882; 0.906)
men	any	≤1 mm	0.990	(0.983; 0.994)	0.982	(0.974; 0.988)	0.969	(0.959; 0.977)	0.951	(0.939; 0.960)	0.933	(0.919; 0.945)	0.868	(0.847; 0.886)
women	any	≤1 mm	0.998	(0.994; 0.999)	0.992	(0.986; 0.995)	0.985	(0.978; 0.990)	0.978	(0.969; 0.984)	0.970	(0.961; 0.978)	0.918	(0.902; 0.931)
any	any	>1-4 mm	0.970	(0.960; 0.978)	0.914	(0.898; 0.927)	0.854	(0.835; 0.872)	0.806	(0.784; 0.825)	0.757	(0.733; 0.778)	0.635	(0.608; 0.660)
men	any	>1-4 mm	0.961	(0.944; 0.973)	0.891	(0.866; 0.912)	0.825	(0.796; 0.982)	0.775	(0.743; 0.804)	0.714	(0.678; 0.746)	0.578	(0.538; 0.615)
women	any	>1-4 mm	0.979	(0.966; 0.987)	0.936	(0.916; 0.952)	0.883	(0.858; 0.905)	0.836	(0.807; 0.862)	0.799	(0.768; 0.827)	0.690	(0.654; 0.724)
any	any	>4 mm	0.863	(0.827; 0.892)	0.700	(0.654; 0.741)	0.549	(0.501; 0.594)	0.462	(0.414; 0.508)	0.406	(0.359; 0.453)	0.286	(0.242; 0.330)
men	any	>4 mm	0.863	(0.813; 0.901)	0.676	(0.613; 0.731)	0.535	(0.47; 0.596)	0.442	(0.379; 0.504)	0.389	(0.327; 0.451)	0.271	(0.214; 0.330)
women	any	>4 mm	0.862	(0.805; 0.904)	0.730	(0.661; 0.788)	0.566	(0.492; 0.633)	0.487	(0.414; 0.556)	0.428	(0.356; 0.497)	0.305	(0.239; 0.373)

Figure S1. Annual age-standardized melanoma incidence rates among men, overall and according to Breslow thickness at diagnosis (≤1, >1-4, >4 mm), and corresponding temporal trends (calculated via joinpoint analysis – see text for details). Tuscany, central Italy, 1985-2017. Only cutaneous melanoma with known Breslow thickness at diagnosis were included.

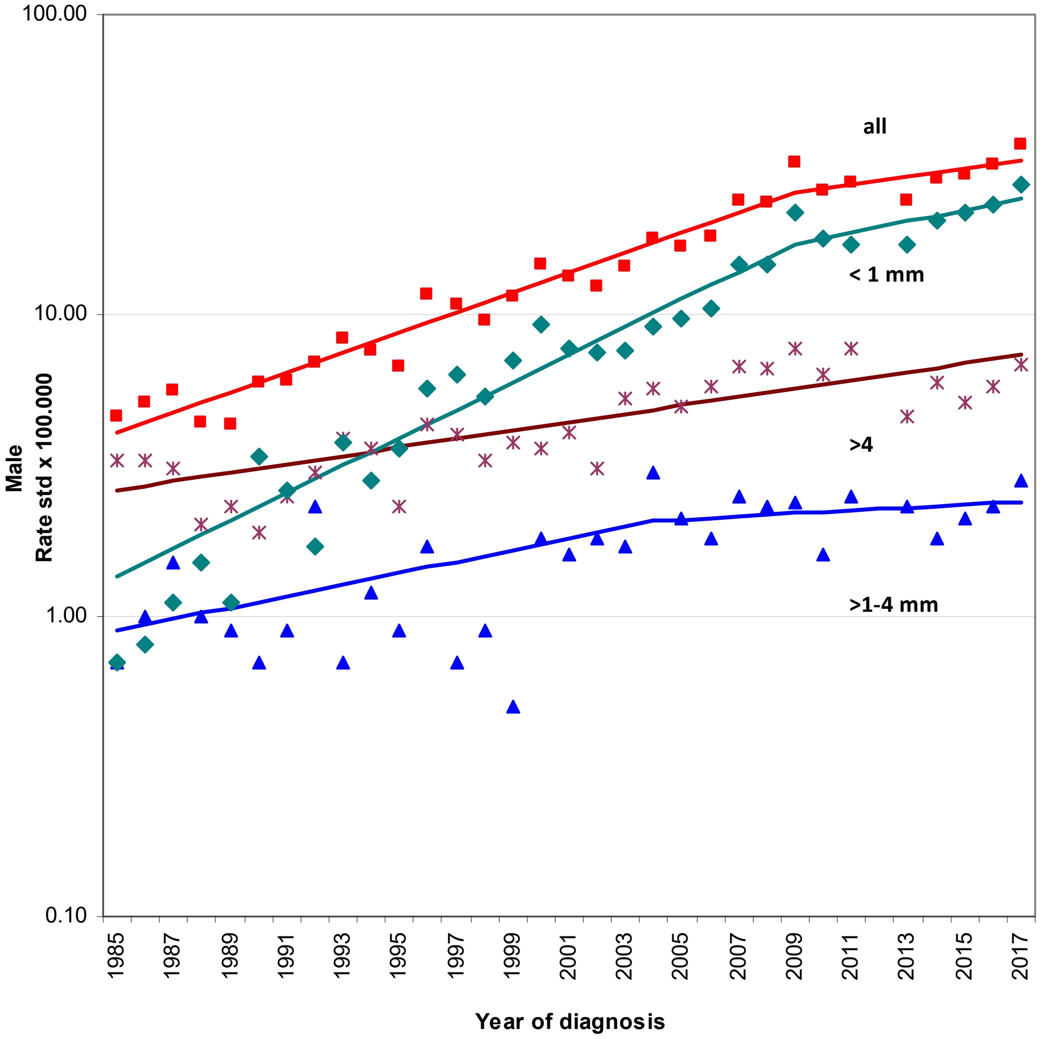

Figure S2. Annual age-standardized melanoma incidence rates among women, overall and according to Breslow thickness at diagnosis (≤1, >1-4, >4 mm), and corresponding temporal trends (calculated via joinpoint analysis – see text for details). Tuscany, central Italy, 1985-2017. Only cutaneous melanoma with known Breslow thickness at diagnosis were included.

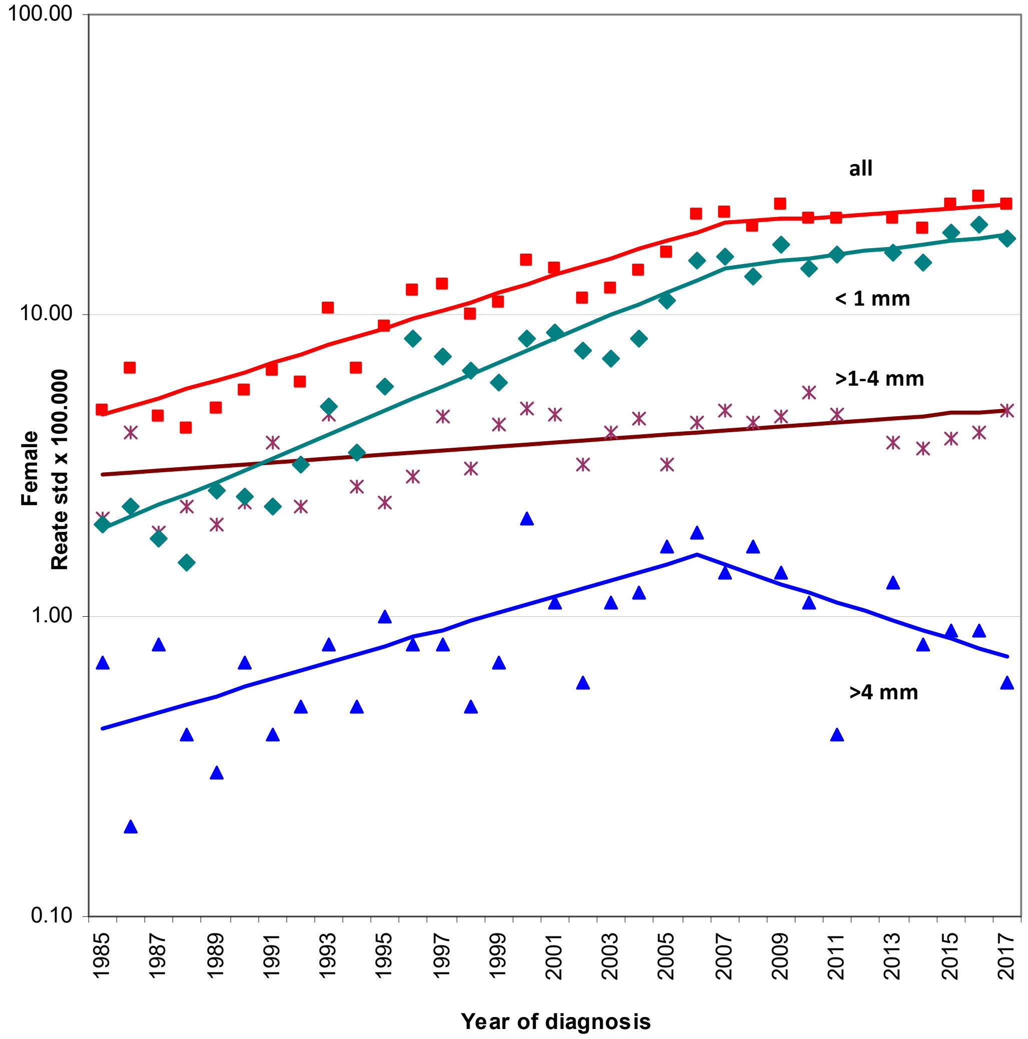

Supplement: Supplementary file 1 [file cancers-16-00536-s001.zip › cancers-2812198-supplementary.pdf]
